# Alcohol brief interventions practice following training for multidisciplinary health and social care teams: A qualitative interview study

**DOI:** 10.1111/dar.12193

**Published:** 2014-09-06

**Authors:** Niamh Fitzgerald, Heather Molloy, Fiona MacDonald, Jim McCambridge

**Affiliations:** Institute for Social Marketing, UK Centre for Tobacco and Alcohol Studies, School of Health Sciences, University of StirlingStirling, FK9 4LA, UK

**Keywords:** alcohol consumption, brief intervention, training, qualitative, social work

## Abstract

**Introduction and Aims:**

Few studies of the implementation of alcohol brief interventions (ABI) have been conducted in community settings such as mental health, social work and criminal justice teams. This qualitative interview study sought to explore the impact of training on ABI delivery by staff from a variety of such teams.

**Design and Methods:**

Fifteen semi-structured telephone interviews were carried out with trained practitioners and with managers to explore the use of, perceived need for and approaches to ABI delivery and recording with clients, and compatibility of ABIs with current practice. Interviews were analysed thematically using an inductive approach.

**Results:**

Very few practitioners reported delivery of any ABIs following training primarily because they felt ABIs to be inappropriate for their clients. According to practitioners, this was either because they drank too much or too little to benefit. Practitioners reported a range of current activities relating to alcohol, and some felt that their knowledge and confidence were improved following training. One practitioner reported ABI delivery and was considered a training success, while expectations of ABIs did not fit with current practice including assessment procedures for the remainder.

**Discussion and Conclusions:**

Identified barriers to ABI delivery included issues relating to individual practitioners, their teams, current practice and the ABI model. They are likely to be best addressed by strategic team- and setting-specific approaches to implementation, of which training is only one part. *[Fitzgerald N, Molloy H, MacDonald F, McCambridge J. Alcohol brief interventions practice following training for multidisciplinary health and social care teams: A qualitative interview study.* Drug Alcohol Rev *2015;34:185–93]*

## Introduction

Alcohol brief interventions (ABI) can be described as relatively short conversations or other efforts that seek to detect people who drink alcohol at a level that is risky or harmful to health and ‘motivate them to do something about it’ [1]. ABIs are heterogeneous, varying in ways including length, content, delivery, deliverer and target group [2].

There is evidence supporting the efficacy of ABIs in reducing alcohol consumption among hazardous and harmful drinkers in primary care settings [3]. While ABI research is emerging in some other healthcare settings [4], little is known about implementation and effectiveness in wider health and social care settings (e.g. mental health, social work and criminal justice settings).

The interaction between alcohol and mental health is complex, and problems manifest themselves in different ways across the spectrum of use [5]. ABIs appear not to reduce drinking in general practice patients with problem drinking and comorbid anxiety or depressive disorders [6]. A systematic review [7] found that the evidence of brief intervention effects in patients with substance use and severe mental health problems was unconvincing.

In children and families social work, concerns about parental misuse of drugs or alcohol are associated with poor outcomes for children [8], and the issues arising can be complex [9]. Assessment of parental substance use by social care professionals has been found to take place ‘often at a late stage with little to no guidance on how to do so effectively’ (p. 1) [10]. One randomised trial involving training for child-care social workers showed that this was challenging [11].

Prevalence rates of alcohol problems among offenders are higher than in the general population 12–14. Studies in England and Scotland identify that clients are positive about receiving alcohol interventions in probation settings [15] and that screening and ABIs are acceptable to practitioners [16].

The duration of training needed to deliver ABIs depends on the complexity of the ABI and the skills of the practitioner. Training for simple brief advice in primary care was delivered in 1 h in a recent major trial [17], though this produced no evidence of effectiveness. The Scottish Government national ABI training was developed as a two day/11 h course [18]. Learning to deliver motivational interviewing has been described as ‘an ongoing process’ in which ‘with practice and feedback you can become more proficient’ [19], though trials of two day workshops have shown no impact on skill acquisition [20],[21].

Inadequate training is one of a range of barriers to the implementation of ABIs. One systematic review found that screening and brief intervention rates ‘generally increased with the intensity of the intervention effort, i.e. the amount of training and/or support provided’ [22]. Another found that ‘adequate resources, training and the identification of those at risk without stereotyping are the main facilitators in primary care’ but that ‘more research is needed to assess implementation in other settings’ [23]. Training and related national initiatives have met with only modest success in securing widespread implementation of ABIs [24],[25].

In December 2007, in advance of publishing their draft national alcohol policy [26], the Scottish Government announced a target [27] for the delivery of ABIs by the National Health Service (NHS). The target comprised projected numbers of ABIs to be delivered by doctors and nurses within primary care, accident and emergency and antenatal settings. One local health service, NHS Greater Glasgow and Clyde, took a strategic decision to implement ABIs across all community health and social care services over and above the priority settings included in the Government target. Managers were encouraged to consider any frontline practitioners for training on ABIs if the practitioners could have a role in discussing alcohol on a one-to-one basis with service users. Create Consultancy Ltd. (owned by N. F.) was commissioned to provide training.

It has been shown that implementation of ABIs is influenced by senior staff and systems as well as individual-level factors 28–30. By interviewing training participants and managers in NHS Glasgow and Clyde, this study aimed to explore how, if at all, training had impacted on practice in relation to alcohol, whether or not ABIs were being delivered and recorded and why.

## Methods

### The training

Nine 1 day training courses were delivered to 89 staff between June 2009 and March 2010. The training course was based loosely on the Scottish Government ABI definition [31] but designed specifically for multidisciplinary staff including health, social care and other practitioners (see Appendix 1). The objectives and methods are provided in Table 1 which, due to the multidisciplinary nature of the group, was not prescriptive about how, when and with whom to raise the issue of alcohol. Participants did not receive an intervention manual but were provided with detailed handouts. The recording system for ABI delivery was established and communicated separately to participants, and no specific supervisory arrangements were made for ABI delivery post-training.

**Table 1:** Aim, objectives and methods of the 1 day training course

Aim: To build on practitioners' existing skills in competently, confidently and appropriately raising and responding to alcohol issues with their clients/patients including delivering brief interventions	
Objectives: After this course, participants will:	Activity and teaching method
Have a basic understanding of the principles of discussing behaviour change with clients in a motivational way	Short paired role play
Have considered their own and others' attitudes to alcohol and how they may impact on providing brief interventions on alcohol in practice	Small group discussion of attitude statements
Have a basic understanding of drinking limits and how to estimate the number of alcohol units in alcoholic drinks	Small group quiz with feedback
Have been introduced to the basic stages involved in delivering a brief intervention on alcohol to clients	PowerPoint presentation
Have reflected on what challenges they perceive in raising and responding to the issue of alcohol with clients/service users using brief interventions where appropriate and how these could be overcome; and on the opportunities that the delivery of brief interventions present for themselves, their service and their clients	Individual reflection and whole group discussion
Have become comfortable with a variety of ways appropriate to their role they could raise alcohol as an issue with clients who would be an appropriate target for a brief intervention	Individual reflection and whole group discussion
Have considered how they would explore levels and patterns of alcohol consumption with their clients accurately but in an objective and non-judgemental way.	PowerPoint presentation and paired role play activity
Have had an opportunity to practise delivering a brief intervention	Observed role play in triads
Have considered what further learning and support needs they have to become competent and confident in delivering alcohol brief interventions to clients	Whole group discussion and individual reflection

### Sample

Purposive sampling was used to select staff for interview from different staff groups prioritising the teams and professions from which attendance was greatest. The sample size was dictated by the resources available and timescale for the study. Within each category, several attempts at contact were made with trainees, and the final sample consisted of nine practitioners and six managers of the 18 originally sought (see Table 2). One team manager declined to have her team identified and is referred to as ‘anonymous team’ throughout. All of the practitioners had attended the training, as had one of the managers. Interviewees were not reimbursed for study participation.

**Table 2:** Distribution of participants in training and study sample by team and profession

Group	Total number of practitioners trained	Practitioner sample selected for interview/sample who took part in interview	Managers selected for interview/managers who took part in interview
Community mental health team 6 Community psychiatric nurses 3 Occupational therapists 3 Health-care assistants 2 Social workers	14	3/3	2/2
Other social work teams 6 Criminal justice 6 Schools based 6 Children and families/early intervention 4 Young people's team 3 Long-term team 14 Others/undeclared	39	4/4	2/2
Primary care 4 Health visitors 3 Other nurses 1 Doctor	8	1/0	1/1
Women's Aid (voluntary sector service for women affected by domestic abuse):	5	1/0	0/0
Community older people's team	4	1/1	1/1
Others 4 Health improvement team (no direct client caseload) 3 School nurses 3 Addiction nurses 2 Anonymous team 2 Day centre officers 4 Miscellaneous 1 Practitioner no longer in area	19	2/1	0/0
Total: 15 of a possible 18 interviews were conducted.	89	12 sampled; 9 interviewed	6 sampled; 6 interviewed

### Data

Interviews were conducted between August and October 2010, between 6 and 19 months after the training courses. Interviews were conducted by telephone by N. F., who had no prior contact with any interviewees. Interviews were semi-structured covering the topics shown in Table 3 and lasted approximately 45 min. The initial topic schedule was developed by N. F., H. M. and F. M. Participants were encouraged to speak freely about their experiences, and questions were not asked verbatim of each participant. Interviews were audio recorded, notes were simultaneously typed during the interviews, and the recordings were used afterwards to complete and correct the notes.

**Table 3:** Overview of interview topics

Practitioners	Managers
Reasons for attending the training	Views on the roles of staff regarding alcohol and ABIs
Impact of training on skills, confidence and knowledge	Awareness of staff attendance on ABI training
Impact of training on practice	Expectations of impact of training on staff skills, knowledge, confidence and practice
Use of system for recording and reporting ABI delivery	Knowledge of and views on the system for recording and reporting ABI delivery
Suggestions for support to improve delivery, recording and reporting of ABIs	Suggestions for support to improve delivery, recording and reporting of ABIs

ABI, alcohol brief intervention.

### Analysis

Interviews were analysed thematically by N. F., initially grouping themes under each of the areas defined by the interview guide. Segments were coded manually, and new codes developed using a simple inductive approach as themes emerged [32]. For example, under the topic ‘impact of training on practice’, inductive themes arose including ‘perceptions of need for ABIs’, with sub-themes of ‘severity of alcohol/other problems’ and ‘lack of need’.

### Ethics

Ethical approval was not required as this was deemed a service evaluation. The findings were initially written up in a report for the funder which is in the public domain [33]. Participants were asked to give full and free verbal consent to participate and assured of confidentiality. To this end, both the wording of all quotations and the description of the practitioner to whom they were attributed were explicitly checked with and approved by participants.

## Results

Few practitioners had delivered ABIs to their clients and therefore had not had an opportunity to use or critique the system established for recording such delivery. Emerging themes fell into three overarching categories: ABI delivery; perception of need for ABIs and appropriateness of ABIs for clients; and fit with current practice.

### ABI delivery

Seven of the nine practitioners interviewed in this study reported delivering no ABIs to their clients, and one other was unsure if she had delivered any. The main explanations given by practitioners for this was that they had not encountered clients for whom delivery would have been appropriate and/or that ABI delivery did not fit with their current practice or role. In contrast to the other interviewees, one practitioner in the Early Intervention Social Work team reported delivering ABIs to her clients. This team provides early intervention with children and families at risk of statutory child protection measures, and this social worker could see a need for ABIs in her clients:

*I work with a lot of parents and women in particular…whose use of alcohol I would say is impacting on their childcare and…really affecting their parenting. A lot of the people I work with have what I could call alcohol problems, but maybe wouldn't recognise it.* (Early intervention social worker A)

She went on to describe how she now recognised opportunities to discuss alcohol, where in the past she ‘*let these opportunities slip by’* because she lacked confidence about *‘how to pick it up without offending*’ the client.

*Somebody might say, ‘oh I slept in last week because I had a wee drink’ or ‘I was out with my pals and I never got the weans* [children] *from school’. I was hoping to take a very brief opportunity like that…I'm using it with ones who would not identify themselves as having alcohol addiction and who are not receiving services… with women who if I went to their house just now and said ‘oh you've got a bit of an alcohol problem’ they'd likely put me out and they'd not let me back in again and that is where I'm finding it helpful. It's taught me to get around that without using that kind of language, or without panicking them.* (Early intervention social worker A)

She described how she approached the ABI once she had raised the issue, reporting that she ‘*felt better about how to do it, a bit more gently, more softly’*.

*…maybe to say ‘ well, is that impacting on your life? Could we talk more about that? Is that different to what used to happen?’ Different ways to try to raise their awareness to the fact that they may well be abusing alcohol more than they did or that it's having more of an impact than it previously had.* (Early intervention social worker A)

### Perceptions of need for ABIs and appropriateness of ABIs for clients

Most interviewees reported that ABI delivery was not appropriate for their client group. Some practitioners and managers reported that their clients had long-standing alcohol problems and needed a level of intervention that was greater than an ABI.

*Most people are coming in with intensive alcohol problems or addiction problems.* (Criminal justice social work manager)

Others saw their role as identifying the signs of an alcohol problem and making onward referrals, which were not defined as ABI.

*…if I was to go in to see a client and I seen the signs of someone with alcohol problems then I would ask if I can refer them to the substance misuse worker.* [Practitioner (anonymous team)]

In the Community Mental Health Team (CMHT), it was also felt that many clients had severe and enduring mental health problems which meant that they were ‘*not well enough’* for ABI delivery. This view was supported by one of the CMHT managers. Others reported that their clients were not drinking enough to merit ABIs. One of these was working with children who were not drinking any alcohol and felt it would be inappropriate in the context of her work to raise the issue of alcohol with their parents.

*You wouldn't just routinely raise it with somebody just because you want to make sure that that's not something that's there. There would have to be something that would make you feel [it was] appropriate to raise the issue of drinking.* (Early intervention social worker B)

The Community Older People's Team participants also felt they had very few clients drinking at a sufficiently high level to benefit from an ABI. One of their client groups is elderly patients who have fallen. The relevance of discussing alcohol with this group was acknowledged by both the manager and practitioner interviewed.

*A large percentage of our clients are fallers but anyone that you're seeing who has had a fall there might have been some alcohol in the picture…common sense would prevail in terms of that alcohol makes people fall…so they [the staff] really did see that this [training on alcohol brief interventions] wasn't a bad idea. It meant that you could offer them something in terms of raising awareness in a more accurate way than probably had been previously.* (Community older people's team manager)

### Fit with current practice

Practitioners' descriptions of their current practice in discussing alcohol with clients suggested more complexity than was initially apparent from their reported lack of ABI delivery. Both the Children and Families Social Worker and CMHT practitioners described giving advice on alcohol consumption to clients and felt that the training had helped with this role, though this was not considered as ABI. In the case of the former, she felt her role was one of reinforcement of messages with clients who are also engaging with specialist substance misuse services.

*I think it just helps me to reinforce it and if they're getting it from me during my weekly visits at home. Sometimes some of my clients are not really engaging as well as they could do with their substance misuse worker so if they get it both barrels if you like, you know I'm able to give them my take on it as well…I'm able to advise them about the units that are involved… which is something that I wasn't able to do before [the training].* (Children and families social worker)

In the CMHT, it was apparent from discussion that practitioners provided a range of alcohol interventions with clients ranging from advice about units and discussion of impact of alcohol on lifestyle and mood to provision of resources.

*Some of the literature that was given out I photocopy that and give them out to people which I think they find quite interesting. So it was well worth doing.* (CMHT practitioner)

In the CMHT, community older people's team and criminal justice social work team, practitioners reported that they already ask about alcohol (e.g. using the CAGE (Cut down? Annoyed? Guilty? Eye-opener?) screening instrument) [34] and that they were unlikely to use the Fast Alcohol Screening Test (FAST) questionnaire [35] covered in the training over and above these.

The paper-based recording system set up for practitioners to report delivery of ABIs was also considered an additional burden that practitioners were ‘*unlikely to complete*’. This was true of the social worker who had delivered ABIs also in that she had not recorded her delivery using the system. She and other social workers reported that they record their observations after each contact with clients and would not use a separate system.

*I would put that on as an obs on my computer when I come back but I would not specifically go and look out your sheet and fill it in…I would put it on as ‘saw Mrs Smith today, talked about whatever, whatever…talked a little about alcohol and blah blah’ and I would close that and that would be me having recorded it.* (Early intervention social worker A)

Despite these views, time was not cited as a major barrier to ABI delivery by any of the practitioners. Two of the six managers did note time pressures on staff.

*[H]ow are we going to manage to include this in what we are already doing in terms of very, very, deep, specific assessment work and also the broad assessment work that we do for our patients?* (Community older people's team manager)

*[Alcohol] is one of a very diverse range of issues that might be around for people so I'm not saying that it isn't relevant because it is relevant but its relevant along with a very, very long list of other things that could be impacting on people across a very wide range of things to do with additional needs, families affected by disability, mental health.* (Early intervention team manager)

## Discussion

Following training, most practitioners reported that they did not deliver any ABIs, primarily because they felt that ABI delivery was not appropriate for their clients. Some teams reported that their clients did not have alcohol problems, and others reported that they had alcohol or other problems that were too severe to be addressed by ABIs.

Apart from some use of the CAGE tool, most practitioners did not use formal screening tools to determine levels of need for ABIs. This may have underpinned some of the failure to recognise hazardous or harmful drinkers in each client group as it makes subjective the assessment of need.

Most practitioners reported some enhancements in knowledge or confidence in discussing alcohol issues with clients following the training. They used this in their existing practice relating to alcohol, which included a range of interventions from simple advice, reinforcement of information provided by specialist addiction services and provision of resources, none of which were seen as ABIs. Practitioners used existing assessment and recording processes to underpin their practice, and these processes had not been adapted to support ABI delivery.

There was little in these interviews to suggest that staff felt that it was inappropriate to deliver an ABI if they identified clients who needed one or that it was an unrealistic expectation given the conversations they are already having with clients. The findings suggest, however, that future efforts to implement ABIs should be based on detailed consideration of current practice in each team or other organisational unit targeted. Adaptations to existing assessments, documentation and recording procedures should be considered in consultation with practitioners and managers to support appropriate screening and ABI delivery. The activities used in any training workshop could then be based on the actual practice change objectives set for staff within specific teams. Individual differences in prior training and learning needs within teams should also be taken into account. Training courses delivered to mixed groups of practitioners from different teams are likely to have their limitations as the barriers identified here are at a team level, rather than being restricted to individual practitioners.

Avoiding an excessively narrow conceptualisation of ABI seems important here. These practitioners see the need for different varieties of ABIs but do not define them in this way. The ABI training model could have more clearly encompassed dual goals (i.e. to motivate self-change and/or help seeking) and sought to enable practitioners to recognise when each is appropriate. Meeting the recognised need to better develop study of ABI content [36],[37] should be accompanied by trials investigating effectiveness in settings such as these, where alcohol problems are commonly encountered, but the literature has been slow to develop [38],[39].

Studies of the impact of training on communication skills development, more generally in healthcare professionals, have noted that training alone may be insufficient to translate skill development into changes in practice [40] without the incorporation of strategies for transferring learning into practice including supervisory support, self-generated feedback and observation of practice [41],[42]. These mirror wider findings on motivational interviewing training 19–21.

The suggestion that training workshops alone may be insufficient to change practice is also supported by a growing body of ‘implementation research’ on how innovations, such as ABIs, are incorporated into routine practice. The Consolidated Framework for Implementation Research outlines 37 constructs or factors which can influence the implementation of innovation [43]. The range and complexity of these potential factors make it unsurprising that the activities outlined here did not result in profound changes in practice for most practitioners. Changing practice requires long-term, relatively intensive effort which is focused on understanding the implications of an innovation for the setting, building support among practitioners and managers, and providing solutions to barriers and challenges specific to that setting [44],[45]. While one of the managers in this study attended the training course, that is just one of a wide range of strategies that may help to support implementation [43].

The early intervention social worker (A) is unlike other interviewees. She was ready to learn about ABIs and was enabled by the training to raise the issue of alcohol and discuss it. Those who implement innovations before others have been described as ‘innovators’ or ‘early adopters’ in implementation research and may be a valuable resource in supporting the design of models of ABI delivery and/or advocating for delivery by others [46]. It should be borne in mind, however, that these findings are based on a limited number of interviews with practitioners and managers, and it cannot be assumed that their views and experiences are representative of others in their team or the larger group who were trained.

Training workshops are not enough to attain routine delivery of ABIs. Subject to trials providing evidence of efficacy or effectiveness, further efforts to implement ABIs in new settings should take time to understand current practice, be aware of levels and types of need in the client group, adapt intervention content accordingly, build support for implementation among practitioners and managers, and provide training as part of a comprehensive implementation plan, constructed so as to be evaluable. ABI programs can only produce the benefits expected of them, if well implemented.
